# Measurement of High Carbon Nanotube Growth Rate, Mass Production, Agglomeration, and Length in a Floating Catalyst Chemical Vapor Deposition Reactor

**DOI:** 10.1021/acsnano.4c15449

**Published:** 2025-02-24

**Authors:** Shahzad Hussain, Joe C. Stallard, Cyprien Jourdain, Michael W. J. Glerum, Jack Peden, Rulan Qiao, Adam M. Boies

**Affiliations:** † Department of Engineering, University of Cambridge, Trumpington St, Cambridge CB2 1PZ, U.K.; ‡ Department of Mechanical Engineering, Stanford University, Building 530, 440 Escondido Mall, Stanford, California 94305-3030, United States

**Keywords:** Carbon nanotube, aerosol, agglomeration, growth kinetics, floating catalyst chemical vapor deposition

## Abstract

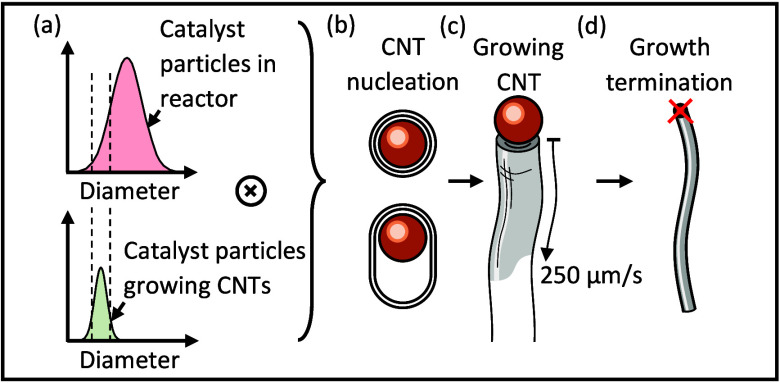

The growth kinetics of carbon nanotubes (CNTs) and precursor pyrolysis mechanisms within floating catalyst chemical vapor deposition (FCCVD) reactors have remained opaque despite significant interest in the catalytic mechanisms, CNT growth, and aerogel formation. This study utilizes in situ characterization of reactants and CNTs to determine CNT growth kinetics. By modulating precursors, we avoid the formation of a CNT aerogel within the reactor, which enables direct sampling at independent axial locations of single and agglomerated CNTs and catalyst nanoparticles. Electron microscopy of the in situ sampled aerosols enables measurement of the length of the nanotubes within them and the extent of nanotube agglomeration. Concurrent real-time individual CNT and catalyst mass measurements via a centrifugal particle mass analyzer details the evolution of individual and bundled CNT masses. The number density of CNT-containing particles increases >10-fold as flow travels through a zone of rising temperature. CNT lengths range from 0.1 to 54 μm, and CNTs of length >10 μm account for over half of the total mass produced. A conservative measure of the CNT mean growth rate of 250 μm/s is the highest growth rate observed in literature. A comparison of experimentally determined CNT growth rates reveals that the exceptionally high rates achieved in FCCVD reactors is due to the uniquely high reactor temperatures (>1500 K). The rate of CNT mass production within the reactor does not vary monotonically with temperature, which suggests that other factors, such as changing activity of catalyst, determine the overall CNT mass production rate.

Floating catalyst chemical vapor deposition (FCCVD) reactor synthesis of carbon nanotubes (CNTs) has received significant attention due to the high rate of production, long resultant CNTs, and formation of macroscopic aerogels. Continuous formation of macroscopic material from the FCCVD reactor distinguishes it from fixed-bed or forest-growth techniques in which a fixed catalyst location prevents continuous removal of product, and endows the process with potential for industrial-scale production.^1,2^ A survey of reported experimental parameters used to manufacture spinnable aerogels^3^ reveals that all provide mean reactor residence times of 5–240 s and operate at temperatures between 1400 and 1800 K. Catalyst precursors often contain iron and sulfur, whose atomic ratio Fe:S varies between 0.1 to 10 across different studies.^3^ A carrier gas is employed to dilute precursors within the reactor, and the concentration of carbon-containing molecules is approximately 3% by total number.^3^ Calculations suggest that gelation of the nanotubes within the reactor can result from collisions brought about by Brownian motion.^4^ In this case, their rate of agglomeration increases with increasing nanotube length and number density within the reactor gas. It is also possible that nanotube agglomerates grow in mass and dimension as additional nanotubes nucleate and grow from adhered catalyst particles upon them; an aerogel then forms as the growing particles impinge. The relative contribution of these mechanisms to aerogel formation remains to be fully detailed.

In-situ observations of CNT growth with electron microscopes^5−9^ show that nanotube growth can initiate when a portion of the carbon layer upon a metal catalyst particle partially detaches and elongates into a short tube. Subsequent growth of the nucleated tube is driven by the conversion of activated carbon gas reactants impinging on the catalyst particle, which transfers the carbon atoms from the catalyst to the elongating CNT wall. Neither the initiation of growth from the particle nor the rate of subsequent tube elongation is spontaneous; energy barriers are associated with both processes, and both demand a sufficient temperature to occur. The increasing rate of CNT elongation with rising temperature measured by observations of growing CNTs indicate that the growth rate following initiation possesses a characteristic energy barrier in the range 0.2 eV/C atom to 3 eV/C atom.^8,10−17^ Numerical predictions have suggested a greater energy barrier on the order of 10 eV or more may be associated with the initial nucleation of growth.^18^ The final length of a CNT varies widely, and can exceed several millimeters.^19−24^ Studies have demonstrated that an increasing electrical and thermal conductivity, mechanical strength, and Young’s modulus of macroscopic CNT materials can correlate with an increasing nanotube length.^25−27^ The final height of substrate-grown CNT arrays varies depending upon the choice of reactor conditions; for example, it can increase when a small quantity of water is injected into the furnace.^9,10,22,28^ Substantial interest now exists in the large-scale manufacture of carbon nanotubes.^29^ The mass production of CNTs achieved within a reactor relates immediately to their number, length, and cross-section and also to the residence time required for their nucleation and subsequent growth. Accurate measures of CNT growth rates are now required to aid the design of appropriate FCCVD reactors with requisite residence times.^1^ In this study, the length of CNTs, the rate of their elongation, and their extent of agglomeration within an FCCVD reactor are measured to further the understanding of how the rate of mass production and the process of aerogel formation are controlled.

Some measurements of the length of CNTs manufactured with FCCVD reactors have been reported in previous literature. A direct method was employed by Koziol et al., who observed direct-spun CNT fibers with a transmission electron microscope. By finding the ratio of CNT ends to total CNT length observed within the images, they inferred a length on the order of 1 mm^30^ where missed identification of ends may give an overestimate of length. Other studies have deduced CNT length from the measured viscosity of FCCVD-grown CNTs suspended within a strong acid^31^ and also by comparison of the relative magnitude of peaks in Raman spectra,^32^ and both of these studies suggest the mean length of FCCVD-grown CNTs lies in the range 1–10 μm. The residence time within an FCCVD reactor is typically below 10 s, whereby for CNTs to reach a length of 1 mm, their rate of growth is at least 100 μm/s.^1,2^ Further refinement of these lower-bounding estimates of the growth rate requires a more direct measurement of the distribution of nanotube length and identification of the time scale over which the growth occurs.

Consider how the number density and length of CNTs could be determined within an FCCVD reactor to determine the rates of their nucleation, growth, and agglomeration. One method is the measurement of nanotubes extracted from different locations within the reactor as aerosols and collected on a substrate. This method has been employed previously to measure the length of FCCVD-manufactured silicon nanowires^33,34^ but is challenging for materials such as CNTs which form bundles that hinder the identification of individual tubes. Second, properties of individual nanotubes or agglomerated CNT particles could be characterized in the form of an aerosol. For example, Zacharia et al.^14,35−37^ have demonstrated the use of an electrical mobility classifier to measure the length of carbon nanotubes downstream of an FCCVD furnace.

In this study, experiments are performed to deduce the length distribution of carbon nanotubes manufactured in the FCCVD reactor and to identify the time scale associated with their growth, the extent of nanotube agglomeration within the reactor, and rate of CNT mass production. In-situ measurements of the number density and size distribution of individual and agglomerated CNT particles along an FCCVD reactor are performed by extracting and measuring aerosolized carbon nanotubes and agglomerated CNT particles with a newly applied centrifugal particle mass analyzer to directly determine the mass distribution of particles/CNTs in real time. Further examination of collected CNT particles with a transmission electron microscope allows for determination of the CNT length distribution and the extent of agglomeration. Collectively, the resultant direct measurements of individual particle/CNT mass and length facilitate calculation of the nanotube growth rates and examination of the early stage CNT bundling processes.

## Results and Discussion

### Production of Aerosolized Particles along the Reactor Axis

To determine the growth rate of carbon nanotubes, a measurement of the residence time for nanotube growth must be obtained. Previous studies have indicated that the majority of carbon nanotube production within FCCVD furnaces occurs in the zones of rising and falling temperature that are located on either side of the hottest portion of the furnace.^38^ To quantify the residence time for growth, we measured the number density of particles present within the reactor as a function of location along the reactor centerline in the first of these two zones in which the reactor temperature increases. We found that the number density of particles present within this zone of the reactor increased by over an order of magnitude within a 40 mm portion of its length and that a maximum in the rate of particle production in this zone was located before the reactor temperature reached a maximum.

**Figure 1:**
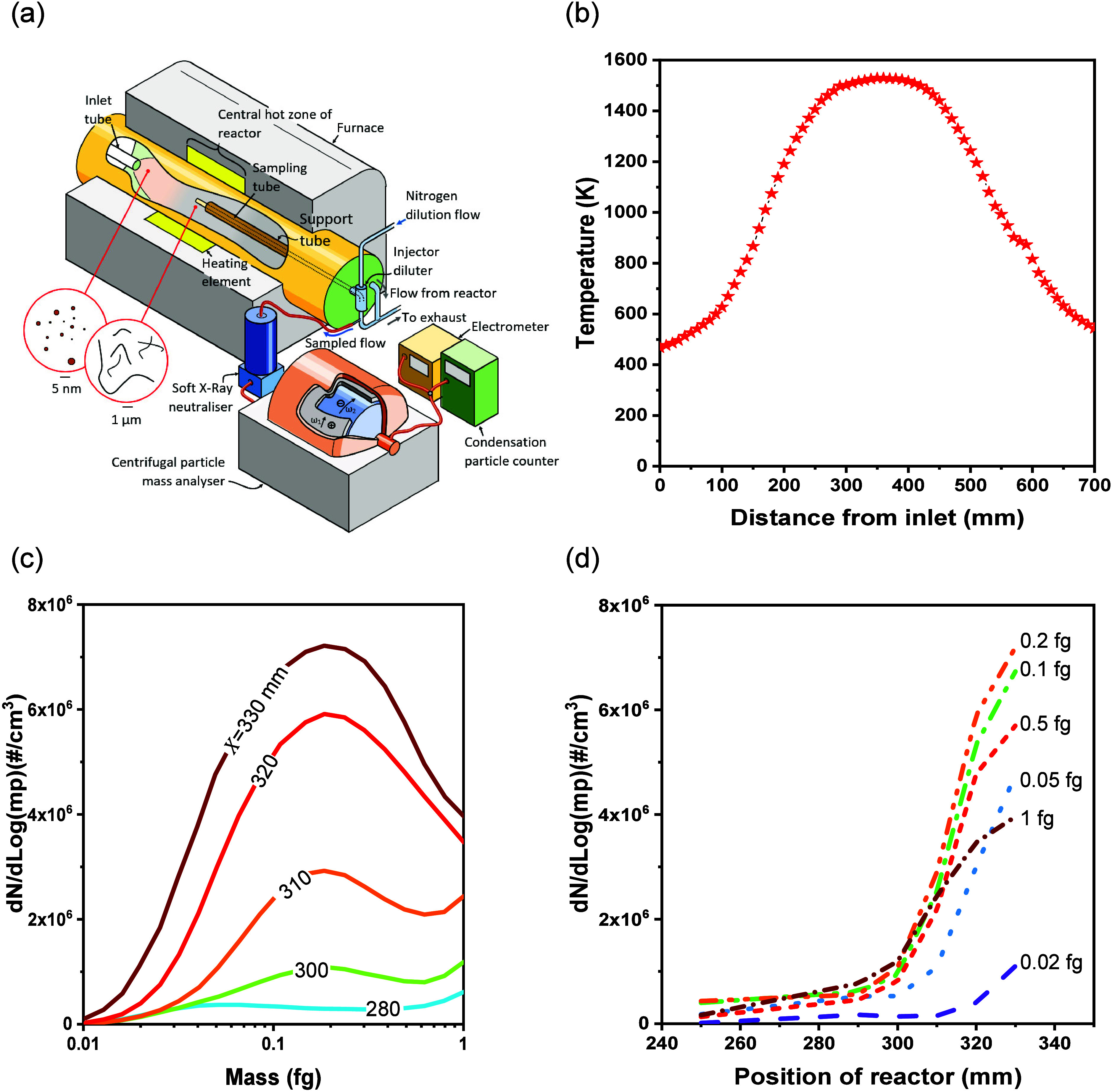
(a) Experimental apparatus for measuring the mass distribution of particles present within the FCCVD reactor. (b) Profile of reactor temperature with distance *X* from tube end. (c) Mass distribution of particles extracted at different positions along the reactor axis in the interval 280 mm ≤ *X* ≤ 330 mm. (d) Evolution of the spectral density for particles of different mass as a function of position *X* along the reactor axis.

A diagram of the FCCVD reactor and experimental apparatus is shown in Figure 1a. Briefly, the reactor comprised an alumina tube (Almath Ltd., Suffolk, UK) which was heated externally within an electric furnace (Vecstar VTF7 3.5 kW furnace, obtained from Vecstar Ltd., Chersterfield, UK). Reactants proceeding along the tube were subjected to the varying temperature profiles plotted in Figure 1b. Ferrocene and thiophene vapors were employed as catalyst and promotor, respectively, and methane as the carbon source. A low flow of ferrocene and thiophene was chosen so that an aerogel did not form within the reactor. This allowed a CNT-containing aerosol to be extracted by a sample probe at different locations along the reactor axis, whose mass distribution and number density of particles could be measured. Sampled gases and aerosolized CNT-containing particles were quenched in nitrogen, and the mass distribution of the particles was investigated with a centrifugal particle mass analyzer,^39^ an electrometer, (Mk. One CPMA and electrometer obtained from Cambustion Ltd., Cambridge, UK), and a condensation particle counter (CPC) (3790 CPC obtained from TSI Ltd., High Wycombe, UK). More details of precursor flow rates and experimental setup are provided in the Materials and Methods section and in the Supporting Information.

Typical spectra of the mass-resolved particle number concentration measured by the CPMA and CPC are plotted in Figure 1c for an assumed unity charge of +1e. For locations *X* ≥ 300 mm, all spectra possess peaks in their mass distribution between 0.1 and 0.2 fg. The magnitude of the particle concentration for a selection of masses is plotted against distance *X* along the reactor in Figure 1d. At all masses, the particle concentration (corresponding to production rate) increases with increasing distance along the reactor. A maximum in the gradient of mass in Figure 1d occurs at between *X* = 300 mm and *X* = 320 mm. Figure 1b shows that temperature (proportional to the speed of flow along the reactor and inversely its average residence time within any interval of given length) increases by less than 2% over the range 300 ≤ *X* ≤ 350 mm. Whereas, the rate of increase in the concentration of particles whose mass lies in the range 0.05 ≤ *m*_*p*_ ≤ 0.5 fg over the interval 320 ≤ *X* ≤ 330 mm is between 8% and 76% below that measured over the previous interval 310 ≤ *X* ≤ 320 mm. This finding is contrary to an expectation that production rate should increase with increasing temperature. Instead, a maximum production rate occurs prior to the peak of Figure 1b.

The plots of Figure 1c are deduced from the CPC counts of particles, which for the range of masses plotted in Figure 1c were in close proportional agreement with the measurement of the electrometer current. From the ratio of CPC count rate and current, it was deduced that each particle possessed a charge of +1e for masses less than about 1.0 fg. For higher masses, the spectra diverged and the electrometer current approached zero, but the CPC continued to indicate the presence of particles for masses up to 10 fg and above (examples of these spectra are included in Figure S2 of the Supporting Information). The existence of nonzero CPC spectra for large masses informs that some large agglomerates do exist within the reactor. This is evidence of some localized CNT aerogel formation, but the recorded counts do not accurately capture the true number density of these larger particles within the reactor.

### Morphology of Aerosolized Particles

Images of particles extracted from the reactor in the sampled flow and collected upon a filter provide insight into their structure and composition at different points along the reactor and allow the typical types of structure present for any given range of particle mass to be determined. It was found that the particles within the reactor were often agglomerates of CNTs, CNT bundles, and catalyst nanoparticles. The diameters and diameter distributions of carbon nanotubes and catalyst nanoparticles were measured from the images as a function of distance along the reactor to characterize their evolution along the reactor axis. This data was used to compute the mass of particles selected according to their mass by the CPMA independently; comparison confirmed that the CPMA accurately selected the nanotube-containing particles according to their mass.

**Figure 2:**
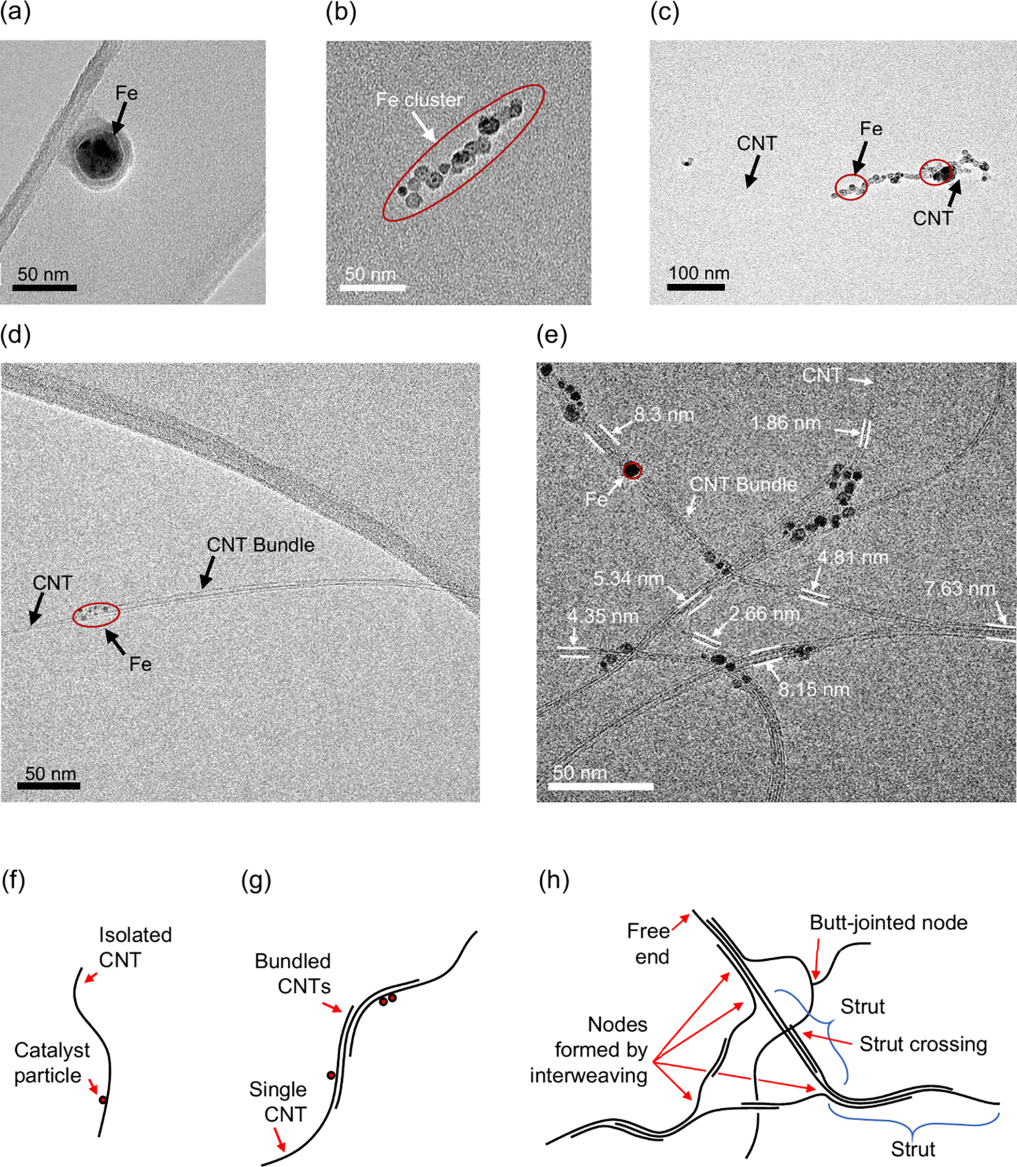
Images of extracted particles obtained with a transmission electron microscope: (a) isolated catalyst particle, (b) catalyst particle cluster, (c) CNT with adhered catalyst nanoparticles, (d) CNT bundle, and (e) CNT cluster; both (d) and (e) have adhered catalyst nanoparticles. Particles extracted from positions (a) *X* = 250 mm and (b)–(e) *X* = 300 mm from reactor tube end. Sketches of CNT particle morphology: (f) isolated CNT, (g) linear bundle, and (h) CNT cluster.

At the position *X* = 250 mm, 110 mm downstream of the end of the inlet tube through which reactants enter, only catalyst nanoparticles were observed upon the TEM grid (Figure 2a). The remainder of the TEM images (Figure 2b–e) of material extracted downstream of *X* = 250 mm are agglomerates of catalyst nanoparticles (Figure 2b), individual nanotubes to which nanoparticles are adhered (Figure 2c), CNT bundles with adhered nanoparticles (Figure 2d), and CNT bundle agglomerates with adhered nanoparticles (Figure 2e). Notably, individual catalyst particles were not found upon TEM grids at locations downstream of *X* = 250 mm, which is consistent with the high collision rates between individual particles and CNTs.^4^

It is instructive to define a taxonomy for the morphology of the CNT-containing particles extracted from the reactor. The CNT particle morphology comprises isolated CNTs, bundled CNTs, and clusters of CNTs, as sketched in Figure 2f–h. CNTs are observed to agglomerate into a linear bundle in which CNTs are adhered and lie parallel to one another. Alternatively, CNTs cluster networks comprise two or more struts that are joined together at the nodes. Within a cluster, a strut comprises one or several bundled CNTs, and nodes are junctions at which three struts connect. The struts have two terminations that can be at nodes, free ends, or a combination of a node and free end. The nodes are classified as interwoven, where CNTs continue from one strut into another through the node, or alternatively as butt-jointed, where one CNT starts its growth from the surface of another, to which its end remains adhered. Nodes are distinct from crossings between struts that are absent of any interweaving of CNTs between the struts. Such crossings may form during the collection of the extracted aerosol upon the TEM grid, as CNT particles deposit upon one another.

**Figure 3:**
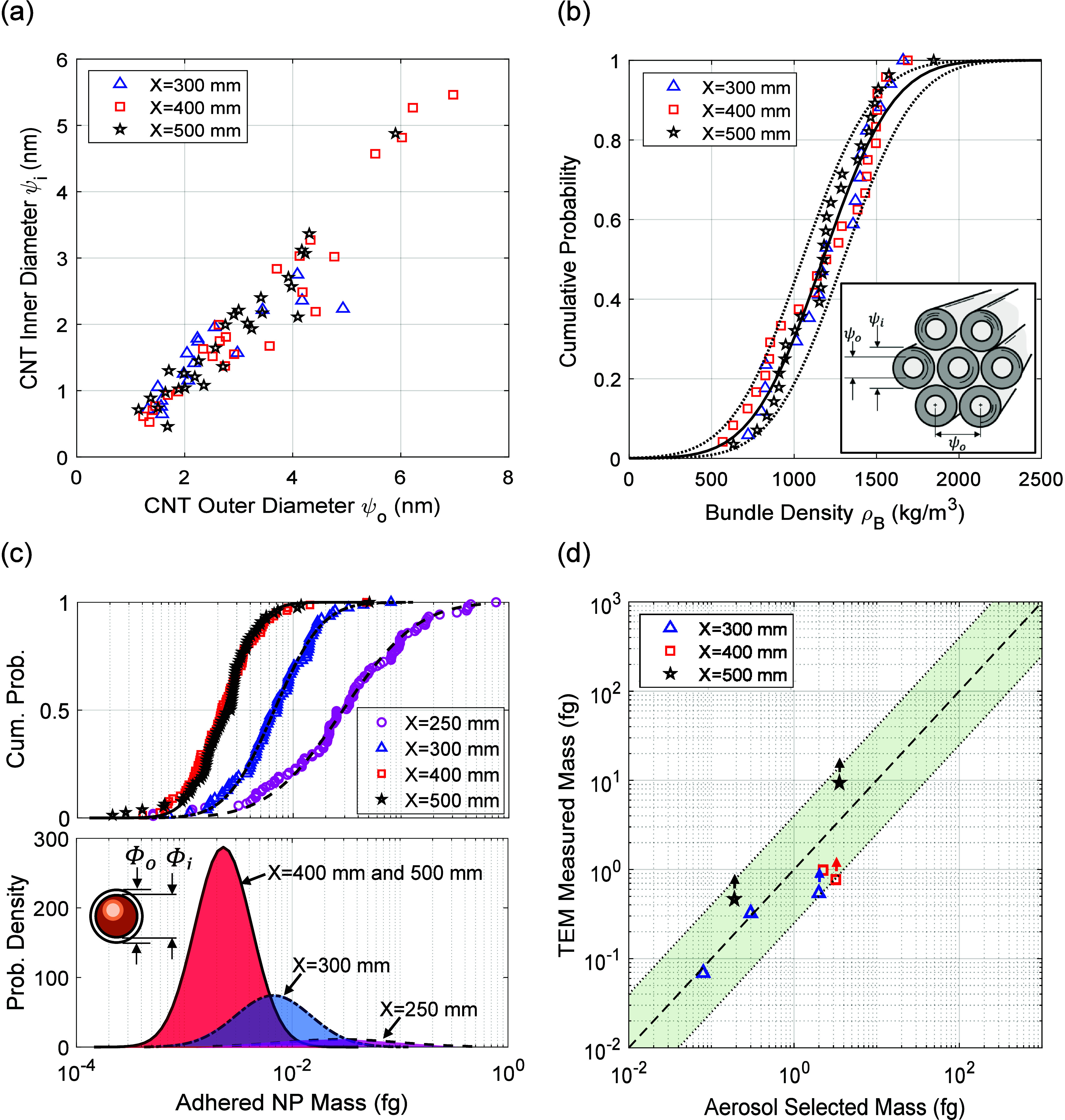
(a) Inner and outer diameters of CNTs as measured from TEM images. (b) Distribution of calculated bundle density based upon hexagonal packing of CNTs (sketched in the inset). (c) Number distributions of catalyst particle mass measured from TEM images for different locations *X* along the reactor axis. (d) Plot of the particle mass deduced from TEM images of aerosolized particles selected by the CPMA according to their mass, where arrows indicate measure is a lower bound.

We then report the mass of the nanotube particles collected from the aerosol. The characteristics of the CNTs and catalyst nanoparticles that comprised the aerosolized particles extracted from different locations along the reactor were investigated by obtaining measurements from the TEM images. CNTs possess characteristic inner and exterior diameters denoted as ψ_*i*_ and ψ_*o*_, respectively. These diameters are plotted for each nanotube in Figure 3a for nanotubes collected at three locations (*X* = 300, 400, and 500 mm) along the reactor axis. The range of inner and outer diameter were 0.5 ≤ ψ_*i*_ ≤ 5.5 nm and 1 ≤ ψ_*o*_ ≤ 7 nm, respectively. A degree of positive correlation was present between ψ_*i*_ and ψ_*o*_, and they rarely differed between 2 nm. The spacing between CNT walls is typically 0.34 nm, and most CNTs possess between 2 and 4 walls. It was not often clear from the TEM images how many CNTs were contained within each strut, so the mass of the CNTs within each network strut was calculated on the basis of an average bundle density, the value of which is sensitive to the assumed dimensions of a representative CNT and the packing of CNTs within each bundle. Here the hexagonal packing^40^ as sketched in Figure 3b was assumed, and the density of the solid CNT walls was taken to be that of graphite ρ_*g*_ = 2260 kg/m^3^.^41^ The average density ρ_*B*_ of a bundle of CNTs with diameters ψ_*i*_ and ψ_*o*_ then follows from the unit cell calculation,
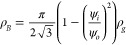
1

A cumulative probability distribution of bundle density based upon the data of Figure 3a is plotted in Figure 3b, and fitted with a Gaussian distribution. Bundle density varies little with position *X* along the axis of the reactor. Its mean value ρ_*B*_ = 1170 kg/m^3^ was employed in the evaluation of the CNT particle mass for all locations. Carbon-coated nanoparticles were taken to be spherical; see Figure 3c. Their mass was calculated from the inner diameter ϕ_*i*_ of the particle and the outer dimension ϕ_*o*_ of the carbon shell as below,
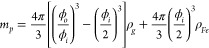
2where the density of the metal in the particles was taken to be that for iron, ρ_*Fe*_ = 7900 kg/m^3^.^42^ The cumulative distribution of the nanoparticle mass by number is plotted in Figure 3c. The average nanoparticle mass decreases with increasing coordinate *X* along the reactor, and the geometric variation of their mass narrows. In all cases, log-normal functions provide accurate fits to the mass distributions and upon differentiation give rise to the probability density functions of Figure 3c. The mass-selected and TEM-derived mass show good agreement (Figure 3d) for variation of CNT and CNT particle masses by over an order of magnitude (0.08 to 4 fg). Where the entirety of a particle could not be observed, it is plotted with an upward arrow on the chart to indicate a lower bound in mass. That all data lies within a range of the CPMA transfer function (bounded by the shaded region by a factor of 4 on either side of the plotted 1:1 line) verifies its accurate operation in selecting particles according to their masses.

### Measurement of CNT Length within Extracted CNT Particles

**Figure 4:**
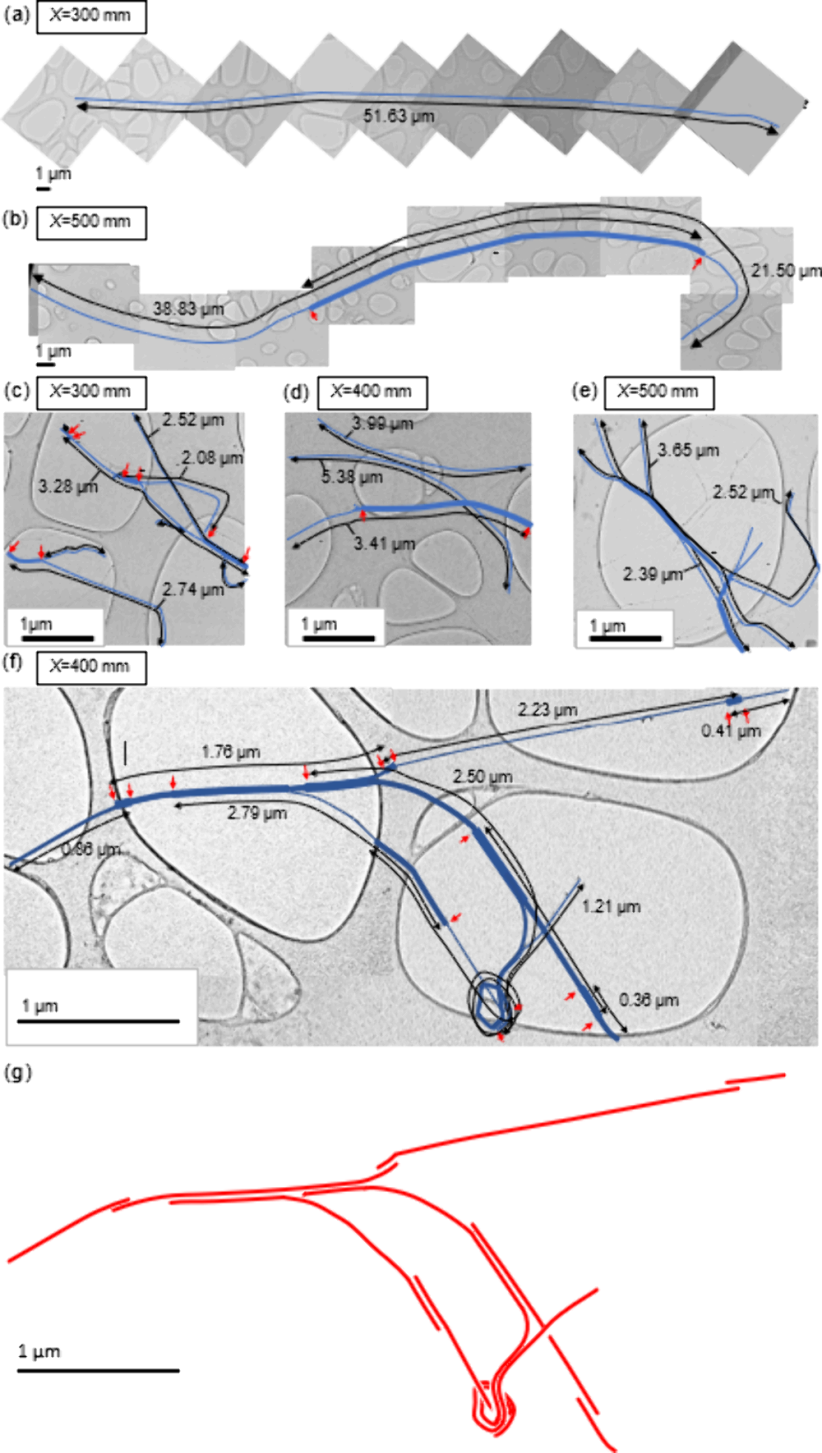
TEM images of CNT particles collected from the reactor. Montages of (a) isolated CNT collected from the position *X* = 300 mm and (b) bundled CNTs at *X* = 500 mm. Images of portions of CNT clusters collected from (c) *X* = 300 mm, (d) 400 mm, and (e) 500 mm. Montage image (f) and map of nanotube paths (g) for a complete CNT cluster collected at *X* = 400 mm.

Of interest to this study is the rate at which CNTs grow within the reactor. To find this, we inspected collected CNT material from the reactor. It was found that the length of CNTs varied between 0.1 and 54 μm. A selection of annotated examples of the measured CNT lengths within collected CNT particles are presented in Figure 4. In the case that a CNT did not bundle with others along any of its length (e.g., Figure 4a), the measurement of length was straightforward. For CNT bundles or clusters, locations of changes in bundle thickness and the geometry of nodes within a cluster were also used to arrive at estimates for length; see Figures 4b–c and Figure 4f where thickness changes are identified with arrows (see methodology in the Supporting Information). An example of an isolated cluster is shown in Figure 4f, and the path that individual CNTs take within the cluster is mapped below in Figure 4g. Notably longer CNTs are more challenging to measure, because it is not always possible to identify their path within clusters. For this reason, it is likely that our results for CNT length that follow underpredict individual CNT length and mass.

**Figure 5:**
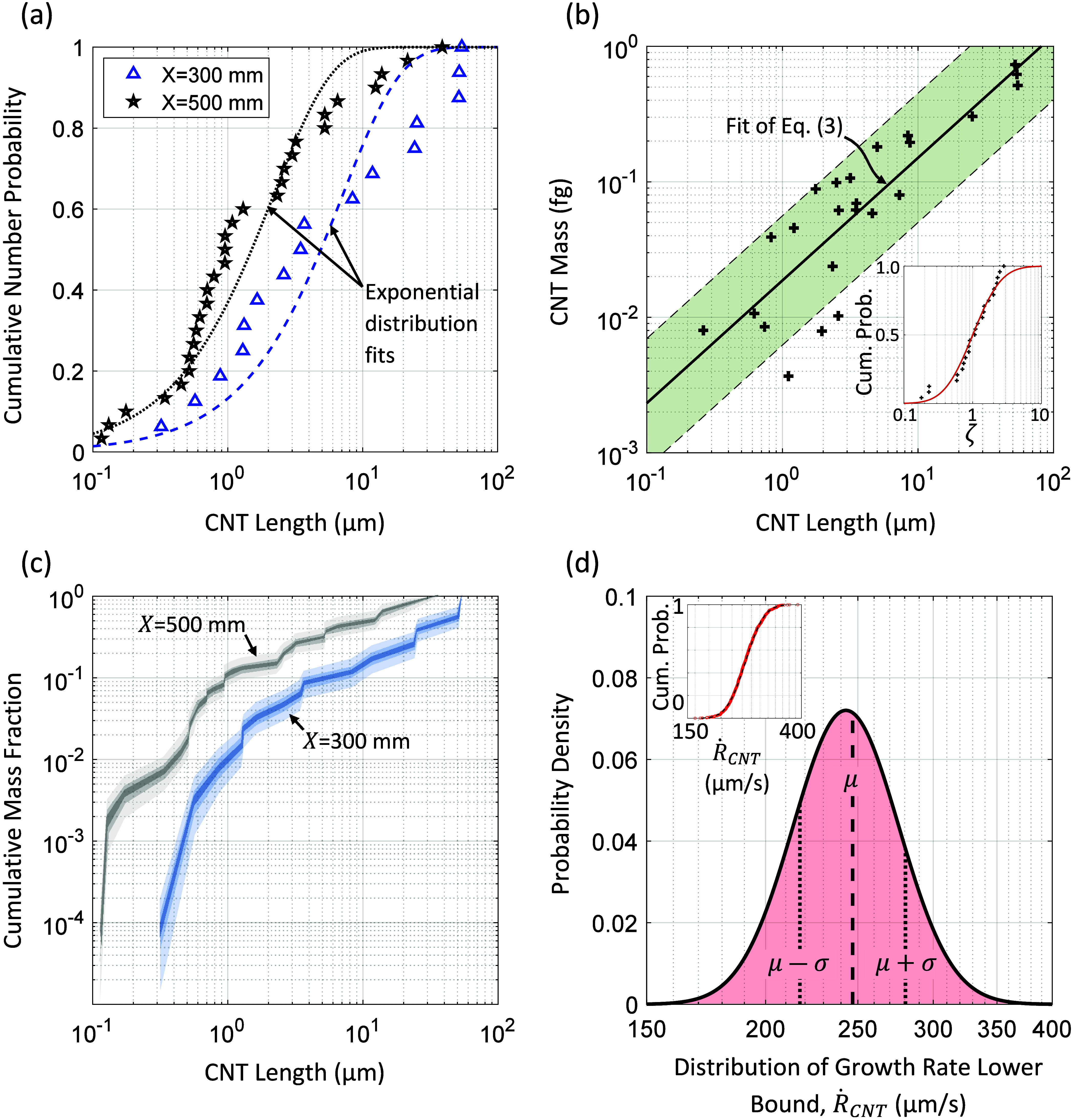
(a) Cumulative distribution of CNT length by number. (b) Plot of calculated CNT mass versus CNT length, including the power-law fit of eq 3 and the distribution of the variable ζ describing the variation of data from eq 3. (c) Family of distributions for cumulative mass fraction versus CNT length for positions *X* = 300 mm and *X* = 500 mm. (d) Cumulative (inset) and probability density of the lower bound for CNT growth rate *Ṙ*_*CNT*_ between *X* = 280 mm and *X* = 300 mm.

Cumulative probability distributions of CNT length by number obtained from the measurement of TEM images are given in Figure 5a for positions in the reactor *X* = 300 mm and *X* = 500 mm. The length of the longest measured CNT exceeded 50 μm and was recorded at *X* = 300 mm. The median length is decreased between *X* = 300 mm and *X* = 500 mm by over a factor of 3, indicating that a greater number of shorter CNTs grow downstream of *X* = 300 mm. The variation in CNT length with position within the reactor may give mechanistic insights for growth. Exponential distributions result from failure due to an event of fixed probability within any time period,^43^ e.g., the failure of a catalyst particle during growth. When exponential distributions are fitted to data of Figure 5a, they lie within a factor of 3.5 of the corresponding lengths of the measured cumulative probability distributions, and thus, FCCVD CNT length may be substantially a function of catalyst inactivation.

Since the measured length of CNTs varies by over an order of magnitude, it is instructive to consider their length distribution not only by number but also by cumulative mass to assesses the relative contribution that CNTs make to the overall mass production as a function of their length. Values of CNT mass *m*_*CNT*_ deduced from the measurement of TEM images are plotted against CNT length in Figure 5b. The CNT mass vs length data are fitted with a power-law relationship, multiplied by a random variable ζ to capture the variance,
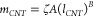
3where the constant *A* = 0.0187 fg/μm and exponent *B* = 0.905. Most data lie within a factor of 3, indicated by the shaded region on either side of the power-law fit *m*_*CNT*_ = *A*(*l*_*CNT*_)^*B*^. The cumulative probability distribution of the variable ζ, obtained by the division of a CNT’s mass by its predicted mass according to its measured length and the power-law fit above, is plotted in the inset of Figure 5b along with a fitted log-normal distribution. The resultant distribution of ζ arises due to the variation in the number of CNT walls and diameter. Other studies^44^ have reported a dependence of the CNT length with CNT cross-section, where the longest CNTs possess a narrow diameter distribution. Here, a minor dependence was observed where exponent *B* is close to unity. CNTs of measured length above 8.5 μm all possessed 3 walls; elsewhere, the number of walls was typically between 2 and 4.

The contribution of CNTs to the overall mass as a function of their length is given by the plot of Figure 5c, which charts the cumulative mass fraction against CNT length with statistical uncertainty included. It is arrived at by using a stochastic algorithm that combines the data of Figures 5a and 5b to give uncertainty bounds, represented in Figure 5c by successively darker regions of shading that capture the central 90%, 50%, and 20% of probability, respectively. To obtain the distributions of Figure 5c, observed values for the variability ζ are picked at random from the set of observed values (inset to Figure 5b) when mass is predicted for each of the lengths plotted in Figure 5a. Successive repetitions of this calculation where mass is summed over all lengths of Figure 5a yield the family of cumulative mass distributions plotted in Figure 5c. For the position *X* = 300 mm, the median tube length by number is approximately 3 μm (Figure 5a), yet it is CNTs of length above 25 μm that account for more than half of the total CNT mass (Figure 5c). This reveals an outsized contribution of the longer CNTs to the overall mass of the product.

### The Growth Rate of CNTs within an FCCVD Reactor

A distribution for a lower bound of the mass-based average CNT growth rate within the reactor is plotted in Figure 5d, whose mean and modal values are close to 250 μm/s. This lower bound is obtained by recognizing that as flow progresses over any interval Δ*X* along the reactor, the fractional increase in total aerosolized CNT mass corresponds, via the cumulative mass distribution of Figure 5c, to a minimum length of CNT that must grow over the interval to account for it. This length, divided by the residence time of gas over the interval, gives the growth rate. A distribution for the growth rate is thus calculated as follows. First, the increase in the total mass of aerosolized particles between the two locations *X* = 280 mm and *X* = 300 mm is found via integration of the spectra plotted in Figure 1c (57%). Then, the minimum length of CNTs for position *X* = 300 mm that accounts for this proportion of total mass is obtained by finding the intercept of a power-law fit constructed within the relevant region of CNT length and mass fraction for each of the family of distributions plotted in Figure 5c. The resulting set of minimum lengths (conservative estimate) is then divided by the residence time of the flow between the two positions (∼0.18 s) to create the cumulative distribution for CNT growth rate *Ṙ*_*CNT*_ that is plotted in the inset of Figure 5d. The calculation of residence time is based upon an assumption of Poiseuille flow along the reactor and described fully in the Supporting Information, supported by finite element simulation. The cumulative distribution is fitted with a log-normal distribution, whose differentiation yields the probability distribution in the main plot of Figure 5d. The magnitude of *Ṙ*_*CNT*_ is on the order 100s of μm/s, and confirms that CNTs grow rapidly within the FCCVD furnace at a rate far exceeding that observed in typical substrate-based growth.^1,2^ The true growth rate of the individual CNTs may exceed the lower bound for the average mass-weighted growth rate that we have calculated; for example, other experiments have found that variations in growth rate of up to an order of magnitude may arise for CNTs of different chirality.^45^

**Figure 6:**
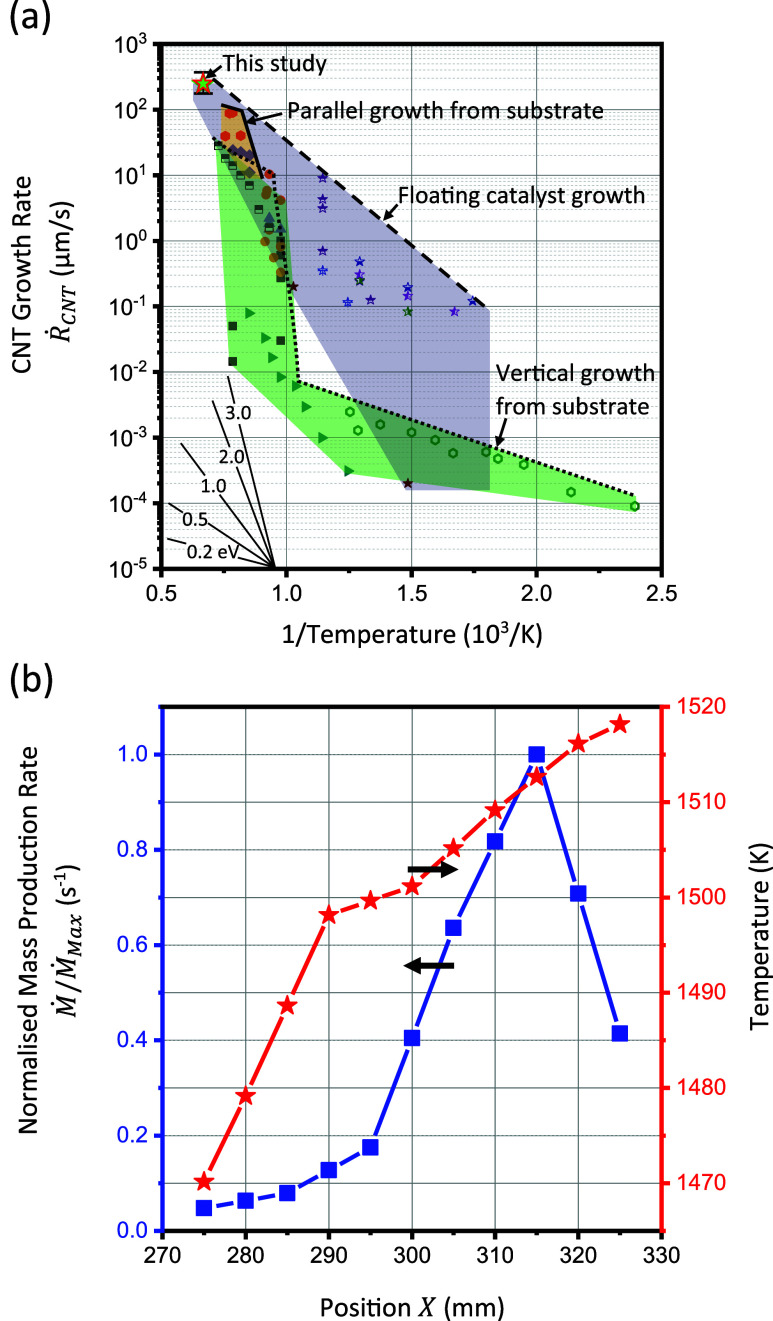
(a) Linear growth rate of CNTs measured in this study and its variation with temperature as reported by other studies in literature.^10,14,19−22,28,44,46−73^ (b) Rate of mass production and rising temperature with increasing *X* in the interval 275 mm < *X* < 325 mm.

An Arrhenius plot of the growth rate measured in this study shows that FCCVD has the highest known growth rate when compared with the measures for a variety of other studies and synthesis techniques, as shown in Figure 6a. The data assembled from the literature is obtained from the chemical vapor deposition growth of nanotubes from carbon precursors over a range of metal catalysts,^10,14,19−22,28,44,46−73^ and includes studies where CNT production is aided by plasma,^54^ the addition of water,^19,21,28,55−61^ and CO_2_ gas.^62−65^ Regions within Figure 6a are shaded to classify three regions of data according to the following types of reactor: CNTs growing vertically from substrates,^19,21,22,28,44,46−59,71−73^ seeding from and growing parallel to a substrate under the influence of flowing gas,^20,22,44,66−70^ and from airborne catalyst particles floating within the gas phase.^10,14^ An overall trend of increasing growth rate with increasing temperature presents for all three classes of reactor, and for any temperature, FCCVD reactors deliver the uppermost growth rates of any reactor type. Gradients corresponding to activation energies in the range 0.2 eV/C atom ≤ *E*_*a*_ ≤ 3 eV/C atom are indicated, within which activation energies measured in individual studies lie; some degree of variation is present for all classes of reactor. Variation in activation energy is attributed by some studies to variables including catalyst chemistry, morphology, and the concentration of precursors.^10,58^ It is also noted by other studies that the activation energy for growth lies in a similar range to that associated with the surface or bulk diffusion of carbon in catalysts.^8,16,17^ For FCCVD CNT synthesis, the gradients of the upper and lower boundaries of the shaded region correspond to energy barriers between 0.63 and 1.46 eV/C atom. The high growth rate measured in this study occurs under higher temperature conditions (∼1500 K for position *X* between 280 mm and 300 mm) than the temperatures employed in other investigations of FCCVD reactors.

### Rate of CNT Mass Production within the FCCVD Reactor

The overall rate of CNT mass production within the reactor is a function of the number of catalysts growing CNTs in addition to the CNT growth rate, inner and outer diameters, and number of walls. The total mass at each location *X* was obtained from integration of the spectra of Figure 1c, and division of the change in mass between the locations by the calculated residence times between them gave the overall mass production rate *Ṁ* as a function of position. It is emphasized that the greatest rate of mass production occurs in the zone of falling temperature downstream of the hottest location within the reactor as shown by Hoecker et al.;^38^ however, here, the production rate is studied in the zone of rising temperature to gain an insight into its evolution along the reactor axis. The absolute mass production rate *Ṁ* is normalized by the maximum mass production rate *Ṁ*_*Max*_ and plotted against location *X* along the reactor axis in Figure 6b to chart the overall reaction dynamics for *X* in the range 275 mm ≤ *X* ≤ 325 mm. The temperature profile in this region is also plotted in aid of the discussion. Overall mass production rate *Ṁ* initially increases with increasing *X*, before subsequently decreasing for *X* in the range 315 mm ≤ *X* ≤ 325 mm. This decrease in *Ṁ* is accompanied by an increasing temperature *T*, and this is contrary to an expectation that the rate of reaction should increase with increasing temperature. *Ṁ* is explored further as a function of reactor temperature in the Arrhenius plot of Figure S5 in the Supporting Information. The effect of temperature upon overall production rate *Ṁ* is charted in Figure S5 and is distinct from the effect of temperature upon the linear rate of nanotube growth; see Figure 6a. Notably, where *Ṁ* increases with diminishing 1/*T* over the range 315 mm ≤ *X* ≤ 325 mm, it does so with apparent energy barrier between 4.5 eV/atom and 95 eV/atom, and this range is distinct from that associated with the linear growth of CNTs which in FCCVD synthesis lies between 0.63 eV/C atom and 1.46 eV/C atom^8^ (recall Figure 6a). The implication of the different effects of temperature upon the individual CNT growth rate and total mass production rate is that other mechanisms or features such as the catalyst particle population, the nucleation of growing CNTs, or the evolving distribution of CNT length control the overall rate of CNT mass production within the FCCVD reactor. Their combined effect endows *Ṁ* with an evolving scaling with temperature along the reactor axis that is distinct to the activation energy characteristic of CNT growth.

In aid of further discussion, a schematic of the CNT growth process is illustrated in the graphical abstract of this article. The process starts with a metal catalyst particle that possesses a carbon coating. Previous literature^10,74,75^ has demonstrated that the diameter of the catalyst particles controls how likely they are to grow a CNT, where the kinetics of CNT growth are determined by size, catalyst chemistry (e.g., sulfur concentration), and CNT chirality. A characteristic energy barrier likely governs the probability of CNT nucleation from candidate catalyst particles of appropriate size.^18^ Once CNT growth initiates, it proceeds with a characteristic growth energy barrier,^8^ and finally terminates at some length whose distribution determines the final CNT mass and which may vary widely with reactor conditions.^9−11,15,21^ The rates of progression between any or all of these stages may affect the rate of mass production within a reactor.

**Figure 7:**
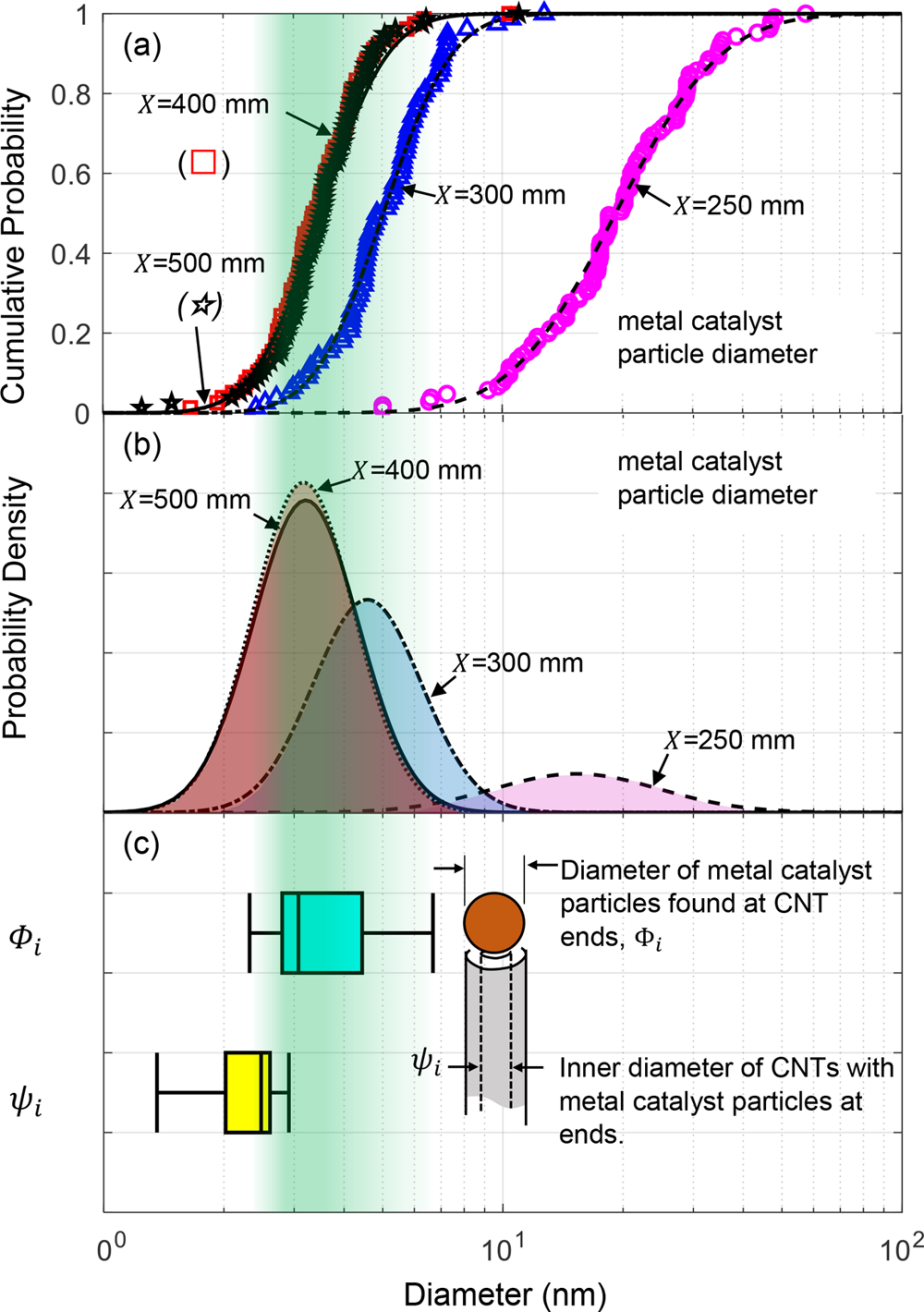
Comparison of (a) cumulative number distribution and (b) probability density of metal catalyst particle diameter with the diameter distributions of (c) catalyst particles found at the tips of CNTs, and the internal diameter of CNTs found with catalyst particles at their tips.

Now consider how the distribution of catalyst diameters, CNT diameters, and CNT length influence the rate of mass production (Figure 7). Recall that the length of extracted CNTs varies along the reactor according to the evolving cumulative mass distribution plotted as a function of the CNT length in Figure 5c. The decrease in length observed between the locations *X* = 300 mm and *X* = 500 mm here acts to decrease the rate of CNT mass production with increasing *X*. Second, consider the effect of the evolving distribution of catalyst particle size. Returning to the catalyst nanoparticle diameter (see Figure 3c), cumulative probability and probability density distributions for the inner metal particle diameters are given in Figures 7a and 7b, respectively. The decrease in particle diameter with increasing distance along the reactor axis matches the trend of previous critical experiments^76^ that have revealed catalyst evaporation and subsequent renucleation are driven by an evolving distribution of temperature (Figure 1b). These diameter distributions are compared with the diameter distribution Φ_*i*_ specifically obtained for the catalyst nanoparticles found at the tips of CNTs, i.e., those catalyst particles from which CNTs grew (Figure 7c). Also plotted is the inner diameter distribution ψ_*i*_ for the CNTs below the catalyst dimensions. For *X* = 250 mm, only <4% of catalyst nanoparticles lie within the range of nanoparticle size Φ_*i*_ observed at the tips of CNTs; this proportion increases by a factor of >20 to 82% and 92% for *X* = 300 mm and *X* = 400 mm, respectively. This increased fraction of particles that correspond to CNT diameters likely contributes to the observed >30× increase in *Ṁ* of Figure 6b. Beyond *X* = 300 mm, the proportion of catalyst in the size range for CNT growth continues to increase. This is accompanied by a decrease in CNT length (recall Figure 5a in which median length decreases by a factor of 5 between *X* = 300 mm and *X* = 500 mm). It is conceivable that this effect contributes to the observed reduction of *Ṁ* in Figure 6b with increasing *T*. What remains to be quantified is the impact of sulfur concentration on the kinetics of CNT growth. Sulfur likely exists as an evolving distribution within the catalyst population, impacting both CNT nucleation and growth. Further, in high-density CNT synthesis, the major fraction of nanotube growth is often obtained in the zone of decreasing temperature that follows the maximum.^38^ In such systems, fresh catalyst particles of greater activity can nucleate from condensation of the metal catalyst vapors from a saturated gas phase, whereby their size lies within the necessary size range to support CNT growth.^38^ We conclude that both the evolving catalyst particle size distribution and CNT length distribution give rise to effects of sufficient magnitude to materially alter the CNT production rate, and so, their influence on the overall mass production likely exceeds that of changing temperature upon the linear growth rate within the zone of rising temperature.

## Conclusions

The growth kinetics of carbon nanotubes within an FCCVD reactor was explored by extracting particles as an aerosol from the interior for online and microscopy analysis. It was found that the length of CNTs could be deduced by the study of images of collected CNTs and CNT agglomerates. Measurements indicated that long CNTs (∼45 μm) grew over a short interval in time (0.18 s). A conservative calculation of growth rate (250 μm/s) showed that the FCCVD-produced CNTs have the highest growth rate in literature. The longest CNTs extracted from the reactor exceeded 50 μm in length. Tubes of length >10 μm account for the majority of the CNT mass, while short tubes result in a much lower number median length of between 0.95 to 3.5 μm. A plot of the growth rate reported here together with the results of other measurements of growth rate within FCCVD reactors^10,14^ and from substrate growth experiments^19,20,22,44,46−59,67−73^ suggest that the growth rate follows an Arrhenius scaling with activation energy in the range of 0.63 to 1.46 eV per incorporated C atom. This barrier is in the range previously reported for substrate growth. Of all CVD reactor types, literature data suggest that FCCVD reactors provide a growth rate above that achieved by growing CNTs from either fluidized beds or fixed substrates.

Mass distributions obtained for the aerosolized CNTs within the FCCVD reactor exhibited a characteristic peak at ∼0.2 fg, whose magnitude rose with increasing distance along the reactor. Catalyst particles were found to adhere to CNTs and CNT clusters. The nucleation of new CNTs and clusters along the reactor is required to explain these spectra, and the mechanism by which this process occurs remains to be fully detailed.

The rate of total mass production within the FCCVD reactor does not follow an Arrhenius scaling with activation energy falling within the range reported for the linear growth of CNTs in the existing literature. Thus, the role of catalyst and CNT length distribution in modulating the rate of the overall CNT mass production were evaluated. The distribution of catalyst particle diameter and number was measured and compared to the size distribution of a subset of catalyst particles that were found to grow CNTs. Although a greater number of catalyst particles are available in the central, hottest portion of the reactor whose diameter lies within the size range employed in CNT growth, and despite an expectation that higher temperature would favor both initiation and subsequent rate of CNT growth, a decrease in the rate of mass production was observed. This decrease is partially accounted for by a decrease in the average carbon nanotube length within the central, hottest portion of the reactor. Overall, effects including the evolving catalyst size distribution, population, and the CNT length distribution account for a much larger portion of the variation in the mass production of the reactor compared to just that of temperature upon the linear growth rate of carbon nanotubes.

## Materials and Methods

The reactor comprised an alumina tube, of length 700 mm, wall thickness 5 mm, and internal diameter 40 mm. The total injected flow of precursors, catalyst, and hydrogen was 0.8 slpm. Methane (CP grade), hydrogen (zero grade), nitrogen gas (UN1066), and argon gas (EC 231-147-0) were obtained from BOC Ltd., Surrey, UK. Ferrocene powder (98%) and thiophene liquid (≥99%) were sourced from Merck Life Science UK Ltd., Dorset, UK. Ferrocene vapor was added to the reactor by passing a 0.015 slpm flow of hydrogen through three copper-lined heated chambers, each of volume 150 mL, with a 30 g mass of ferrocene powder, and maintained at temperature 80 °C. Thiophene vapor was introduced to the reactor by passing a 0.01 slpm flow of hydrogen injected through a porous stainless steel gas diffuser immersed in a 200 mL volume of thiophene. The thiophene was contained within a sealed glass bottle filled with hydrogen. The temperature of the thiophene was maintained at 1 °C by placing it within a thermally insulated ice-filled flask. In each experiment, the reactor tube was heated to temperature by the furnace under a 1.5 slpm flow of air. Each experiment was preceded by flowing purging the reactor tube with a flow of argon (1.5 slpm) for a period of 20 min. The flow was then switched to hydrogen (1.5 slmp) for 20 min. Then, the ferrocene container was purged with a 30 sccm flow of hydrogen for a period of 45 min before the flows above were set for the duration of experiment.

Reactor gases and aerosolized particles were sampled via the extraction of a 50 sccm flow through an alumina sampling tube of inner diameter 1.5 mm, inserted from the downstream end (see Figure 1a). The average residence time of the particles during extraction was much less than the average residence time of flow within the reactor. The flow of sampled gas was quenched upon entry to an ejector diluter value with a 5 slpm flow of nitrogen. Aerosolized particles in a 1 slpm flow of the diluted sampled gases were charged with a soft X-ray neutralizer (model 3087 obtained from TSI Instruments Ltd., High Wycombe, UK), and passed through the gap between the two rotating cylinders of a CPMA. The CPMA allows mass selection of particles/agglomerates from 10^–3^ to 10^4^ fg (8 to 2000 nm at 1 g/cm^3^ density) with resolutions typically between *R*_m_ = 0.1 to 100.^39^ In brief, it operates by subjecting aerosolized particles to a combination of centrifugal force and electrical force as they pass along the gap between two concentric cylinders rotating at different speeds. The mass:charge distribution of particles within the aerosol is obtained by detecting the concentration of particles that pass through the CPMA with either an electrometer or a CPC while the ratio of centrifugal and electric force applied to the aerosolized particles in the CPMA is varied.

Particles were collected from the quenched sampled flow for inspection with a transmission electron microscope (FEI Talos F200X G2 TEM, obtained from Fisher Scientific UK Ltd., Leicestershire, UK). This was achieved by passing a 2 slpm portion of the diluted flow sampled from the reactor through a PTFE filter on which a TEM grid was positioned for particle collection. Particles were collected for a duration of between 2 and 20 min, with a shorter collection time allowing CNT particles to deposit separately from one another upon the grid so that their size could be more easily determined by TEM.

CNT masses were calculated on the basis of their measured inner and outer diameters *ψ*_*i*_ and *ψ*_*o*_, and their lengths *l*_*CNT*_, all obtained from TEM images. The solid walls were assumed to have density ρ_*G*_ of a solid graphite crystal,^41^ so that the CNT mass *m*_*CNT*_ follows as
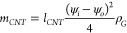
4

## Supporting Information Available

The effect of catalyst concentration and methane precursor upon the formation of aerogel within the reactor. The mass spectra of particles from the reactor as measured with the CPMA for a wide range of particle masses at different locations along the reactor. The method for measuring the length of carbon nanotubes within the agglomerated nanotube particles. The calculation of the residence time of extracted particles within the reactor using a combination of analytical prediction and computational fluid dynamics software. The evolution of the rate of mass production within the reactor as a function of temperature along the axis of the reactor. (PDF)
