# Supplementary material for: Measurement of High Carbon Nanotube Growth Rate, Mass Production, Agglomeration, and Length in a Floating Catalyst Chemical Vapor Deposition Reactor
Source: ACS Nano. 2025 Feb 24;19(9):8739–52. doi: 10.1021/acsnano.4c15449 (PMC11912582; doi:10.1021/acsnano.4c15449)
Supplement: Supplementary file 1 — nn4c15449_si_001.pdf [file nn4c15449_si_001.pdf]

Supporting information for

Measurement of high carbon nanotube growth rate, mass production, agglomeration and length in a floating catalyst chemical vapour deposition reactor

Shahzad Hussain†, Joe C. Stallard†, Cyprien Jourdain†, Michael W.J. Glerum†, Jack Peden†, Rulan Qiao†, Adam M. Boies†,‡,*.

†Department of Engineering, University of Cambridge, Trumpington St. CB2 1PZ, UK.
‡Department of Mechanical Engineering, Stanford University, Building 530, 440 Escondido Mall, Stanford, CA 94305-3030, USA.
*Corresponding author: Adam M. Boies. Email: aboies@stanford.edu.

EFFECT OF REACTOR CATALYST AND PRECURSOR FLOWS UPON AERGOEL FORMATION

Often the ratio of gases injected into FCCVD reactors are selected so as to form an aerogel of CNTs within the reactor. Such an aerogel can be drawn continuously through a gas exchange valve, condensed into a fine fibre, and collected onto a rotating drum. Intending to measure the rates at which CNTs grow and agglomerate, it was necessary to decrease the density of product so that an aerogel did not form, which allowed the CNT aerosol to be extracted from the reactor through a sampling tube. To achieve this, the ratios of injected precursors (methane, ferrocene and thiophene) to the flow of hydrogen carrier gas was reduced successively to discover the limit at which the aerogel did not form. The molar fractions of methane, thiophene and ferrocene relative to hydrogen that were investigated in these experiments are plotted in Figure S1a. In each experiment the methane, catalysts and promotor were injected into a preheated reactor for a period of 15 minutes within a 0.8 slpm flow of hydrogen. The limit of aerogel formation was determined by observing if a CNT aerogel

accumulated at the downstream end of the reactor or alternatively if the CNT media exited the reactor along with the exhausted gas flow: in this case CNTs were found as a dark deposited film upon a filter for the reactor exhaust gases that was otherwise found to be clean when inspected. Images of an accumulated aerogel and clear filter obtained with an injection of methane at a molar fraction of 9.5% are compared with a CNT-covered filter resulting from a more dilute flow of precursors (5.3% molar fraction of methane) in Figure S1b. The observed presence of product within the reactor or downstream on the filter are plotted in Figure S1c: A transition is evident as the flow of methane decreases from 6.35% to 5.3% together with the other precursors: the lowest plotted conditions of Figure S1a were adopted in the subsequent experiments to avoid the formation of an aerogel. Images of the collected nanotube material within the reactor and downstream upon the filter were obtained with a scanning electron microscope (SEM) (TESCAN MIRA3 FEG-SEM, Tescan-UK Ltd., Wellbrook Court, Girton, Cambridge, CB3 0NA, UK), see Figure S1d.

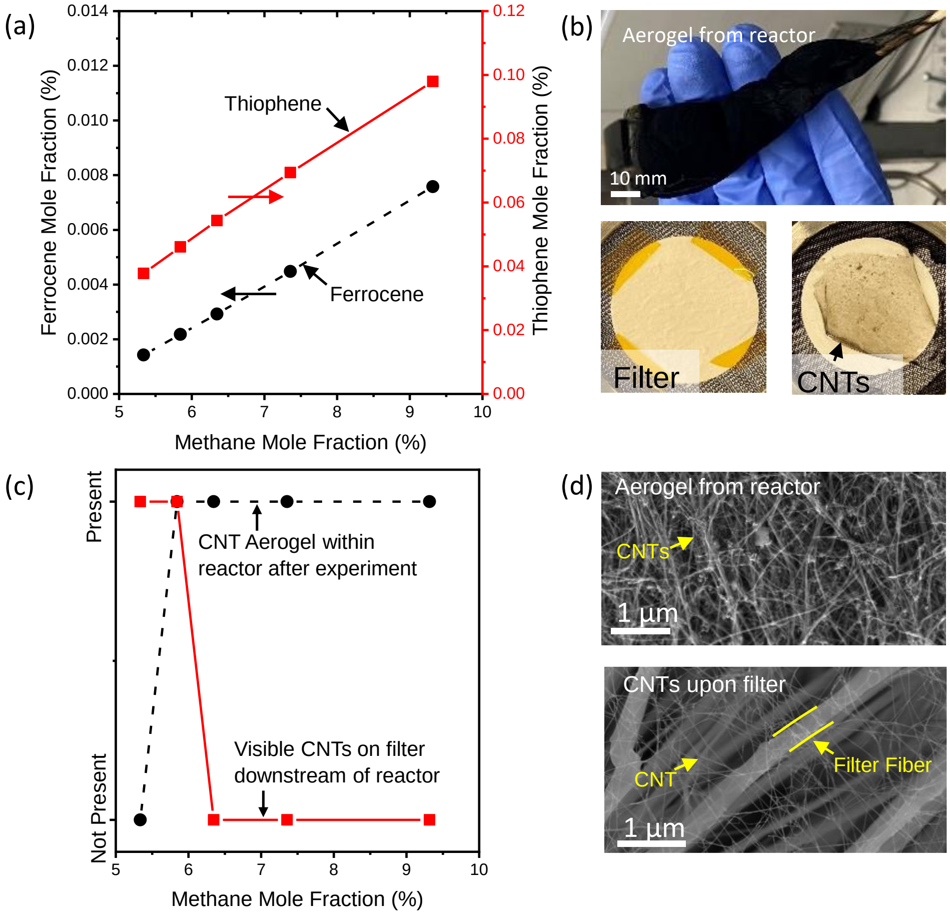

Figure S1: (a) Investigated mole fractions of injected thiophene, ferrocene and methane relative to hydrogen flow. (b) Optical images of a CNTs in the form of an aerogel from the reactor and upon a downstream filter. (c) Observed presence and (d) images obtained with SEM of CNT material deposited within and downstream of the FCCVD reactor.

CPMA SPECTRA PLOTTED OVER RANGE OF LARGER MASSES

Spectra obtained with the CPMA over the range of masses $0.001 \leq m_p \leq 1000$ fg are plotted in Figure S2 as a function of position X along the axis of the reactor for X = 250 mm, 300 mm, 400 mm and 500 mm. Recall that peaks present for masses >> 1 fg far exceed the true number concentration of particles within the reactor that are present for those masses (as is explained in text of the main paper). One explanation is that a CPC fails to accurately count CNT particles of high aspect ratio and size (up to 100 µm), instead interpreting them as a series of many particles in a short time-frame: This was evidenced by a high ratio of CPC counts to electron counts obtained with the electrometer, and the grouping of several CPC counts together as sequences of counts in quick succession.

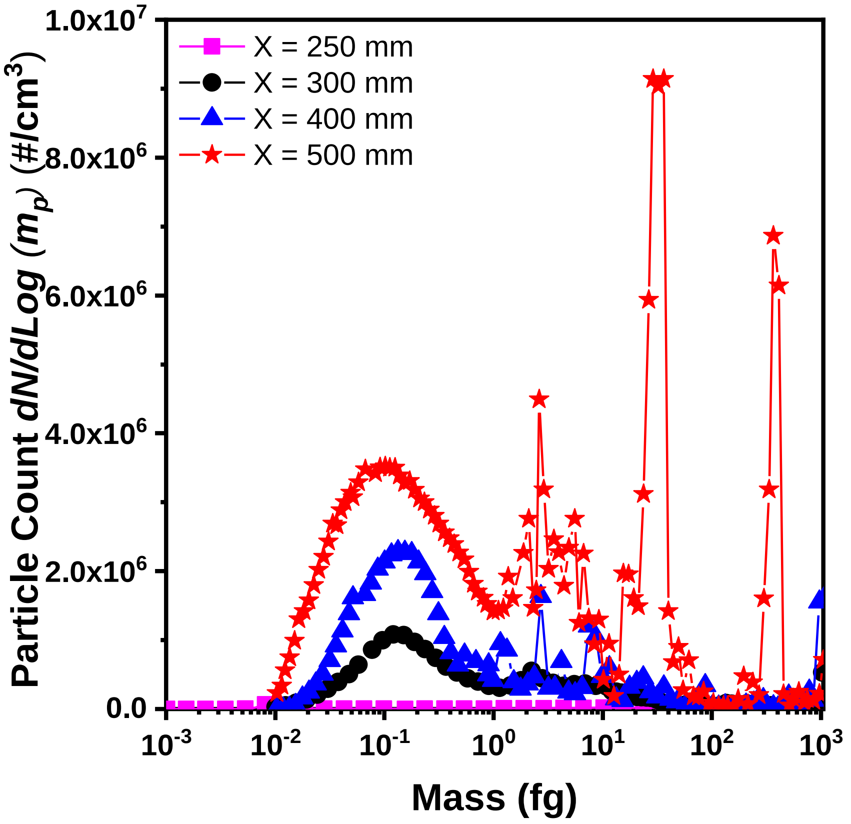

Figure S2: Spectra obtained with CMPA and CPC for different positions along the reactor axis for masses in the range 0.001 fg $\leq m_p \leq$ 1000 fg.

MEASUREMENT OF CNT LENGTH WITHIN EXTRACTED PARTICLES

The length of CNTs within the extracted CNT particles was measured by analysing images obtained from a transmission electron microscope. A series of images annotated to show the method of analysis are presented in Figure 3(a-g) in the main paper. The method for measuring the length of CNTs is as follows:

- Analysis starts from either a free end (where a single CNT forms a strut), or within any strut where the cross section has reduced to a single CNT.
- The CNT is followed from this location along the contour of the strut, and every increase or decrease in thickness of the strut is recorded. Each increase or decrease in strut thickness is interpreted as the location of the start or end of a CNT respectively.
- As interwoven nodes are encountered along the strut being followed, the analysis incorporates them. Nodes connect three struts. Two of these struts are termed minor struts, and they combine at the node into the major strut. Major and minor struts are distinguished by realising that the angle formed between the two minor struts as they enter the node is below that formed between either minor strut and the major strut. It is assumed that the number of CNTs is conserved through a node, so that the sum of the number of CNTs in both minor struts equals the number of CNTS within the major strut.
- Analysis proceeds until any route followed along struts sequentially from the start of the analysis reaches either a free end or a strut whose cross-section is only a single CNT.
- A set of lengths are then obtained by measuring contour distances along the struts between increases and decreases in strut thickness. Beginning at a single location at a free end or within strut where it comprises one CNT, the network is followed along the struts from this point until a decrease in strut thickness closest in its contour distance from the starting location is found: the distance between these locations measured along the network is taken as a CNT length. Then, again from the initial location, the network is again followed along the struts to find the nearest

increase in strut thickness, and the next nearest decrease: this is the second CNT length. This continues until all locations of thickness increase and decrease are accounted for, and the resulting set of lengths are the estimates for CNT length.

It is emphasised that whilst the mean length of CNTs deduced via this method is the true mean length of the CNTs under study, the measure of the longest length is a lower bound. For this reason the uppermost length within the set is a conservative, lower estimate of the greatest CNT length that may exist within the sampled portion of CNT particle.

CALCULATION OF RESIDENCE TIMES IN THE FCCVD REACTOR:

The sampling tube draws a portion of flow from the reactor, and it is concentric with the central, longitudinal axis of the reactor, held precisely in this position by its mounting within the flange that seals the downstream end of the reactor tube. The sampled flow rate $\dot{Q}_S$ of 50 sccm comprises a small fraction $f_s = 0.0625$ of the total reactor flow of $\dot{Q}_R = 0.80$ slpm. Gas flow along the reactor is driven by a minor pressure gradient, and is stationary at the reactor walls. A Poiseuille velocity field develops in which the axial velocity $U(r)$ is a function of radial distance r from the centreline, as written below:

$$U(r) = 2\overline{U}\left(1 - \left(\frac{r}{R}\right)^2\right), \tag{S-1}$$

where R is the radius of the reactor tube and $\overline{U}$ the average axial velocity of flow over the reactor cross-section. Now consider the flow that is sampled. Recognising that the sampling tube is fixed on the longitudinal axis of the reactor and understanding that flow within it is laminar, sampled gas is drawn from a volume enclosed within the radial distance $r_s < R$ from the central axis. Write the sampled flow rate $\dot{Q}_S$ as the integral of velocity over the cross-sectional area within r_s of the centreline,

$$\dot{Q}_S = \int_{r=0}^{r=r_s} U(r) \cdot dA \tag{S-2}$$

$$= 4\pi\overline{U} \int_{r=0}^{r=r_s} \left(r - \frac{r^3}{R^2}\right) \cdot dr \tag{S-3}$$

$$= 4\pi\overline{U} \left[\frac{r_s^2}{2} - \frac{r_s^4}{4R^2}\right]. \tag{S-4}$$

Then write that $Q_S = f_s Q_R$ and that $Q_R = \overline{U}\pi R^2$. Both of these relations are substituted into (S-4) and the resulting expression rearranged to yield a quadratic equation for r_s,

$$r_s^4 - 2R^2 r_s^2 + R^4 f_s = 0, \tag{S-5}$$

whose root $r_s = R\left(1 - \sqrt{1 - f_s}\right)^{\frac{1}{2}}$ satisfies a necessary condition that $r_s \leq R$ for $f_s \leq 1$. Here, substitution of numerical values yields that $r_s = 3.56$ mm, so that $r_s/R = 0.178$.

Now consider the distribution of the residence time τ of the sampled flow located within r_s of the reactor centreline over an increment of the reactor tube length ΔX. The perfect gas law must be applied to recognise the expansion of the flow upon heating to temperature $T(X)$, as the velocities $\overline{U}$ and $U(r)$ discussed so far relate to flow rates $\dot{Q}_R$ and $\dot{Q}_s$ that are reported at the standard temperature $T_0 = 273$ K. Correction for the temperature $T(X)$ as plotted in Figure 1(b) of the main paper gives the true velocity $U(r, X) = U(r)(T(X)/T_0)$, and so the residence time τ follows as

$$\tau(X, r) = \frac{\Delta X}{U(r)}\left(\frac{T_0}{T(X)}\right). \tag{S-6}$$

Now take the sampled gases to be drawn from the area of a 2D plane orthogonal to the reactor axis located at position X, within the distance r_s of the reactor centreline. In this area, r varies over the interval $0 \leq r \leq r_s$, and associated with these limits are respective minimum and maximum values of residence time over the increment ΔX. The ratio of maximum to minimum residence time follows from the manipulation of (S-6) and (S-1) as

$$\frac{\tau(X, r = r_s)}{\tau(X, r = 0)} = \left(1 - \left(\frac{r_s}{R}\right)^2\right)^{-1}, \tag{S-7}$$

and here this ratio has approximate value 1.03. It is immediately apparent that the sampled flow experiences an almost uniform residence time that is close in magnitude to the minimum along the central axis. Here, the conservative upper value of the residence time is employed in the growth rate calculations.

It is also important to clarify any role that the radial diffusion of nanotubes could play: suppose long CNTs were to grow in a zone of greater residence time nearer the reactor walls and then diffuse into the gas volume along the centreline that is sampled: in this case our calculation of representative residence time τ would be an underprediction. However we understand such radial diffusion can be

reasonably neglected, as follows. The diffusion constant associated with a nanotube D relates to its friction factor η and temperature T via $D = k_b T/\eta$; the friction factor η increases (and D decreases) the greater the CNT length. Nanotubes of mass ≫0.01 fg (see Figure 1(c)) possess friction factors $\eta \gg 2 \times 10^{-13}$ kg/s (4), and for the greatest value of temperature $T = 1530$ K have diffusion constants $D \ll 2 \times 10^{-7}$m^2/s. Now over any residence time τ diffusion occurs over the length of order $\delta \sim \sqrt{D\tau}$, which for our $\tau = 0.18$ s gives $\delta \ll 0.2$ mm. Consequently $\delta \ll r_s$, and δ decreases with increasing CNT length. It follows that any effect of radial diffusion of CNTs within the reactor is safely neglected.

To affirm our expectation of how residence time within the reactor is controlled by varying the position of the end of the sampling tube, we have performed finite element simulations using commercially available software (Comsol Multiphysics software, as supplied by Comsol Ltd., Park House, Castle Park, Cambridge, CB3 0DU, UK), to obtain a 3D analysis of the flow field, using hydrogen as the working fluid. The velocity of flows through the reactor inlet and along the exit of the sampling tube were those employed in experiment, and the measured temperature profile along the centreline of the reactor was imposed upon the boundary so as to recreate the condition of experiment. A 3D idealisation was chosen so as to capture the effect of any convection within the reactor, and the effects of gravity were included.

A plot of predicted temperature along the central axis of the reactor against residence time is plotted in Figure S3. Predicted profiles of temperature are obtained for the case that no sampling is performed, for when flow sampled at a positions $T/T_{Max} = 0.98$, and 20 mm upstream of this position: This simulates sampling at the positions $X = 300$ mm and $X = 280$ mm respectively. Upon entry to the sampling tube, sampled flow is cooled due to conduction of heat along the sampling tube. Sufficient pyrolysis of methane is required for nanotube growth. As pyrolysis does not occur below a temperature of approximately 1050°C,[3] once flow cools to this temperature, further production of nanotubes is expected to be minor. Our predicted temperature profiles of Figure S3 inform that flow entering the sampling tube cools below this temperature in < 0.1 s. When the sampling tube is

translated from $X = 280$ mm to $X = 300$ mm, the additional time taken for the flow to reach 1050°C is confirmed to within 1% of the value as calculated above. From this it is clear that the residence time of sampled gas within the reactor is controlled by variation of sampling tube position as anticipated.

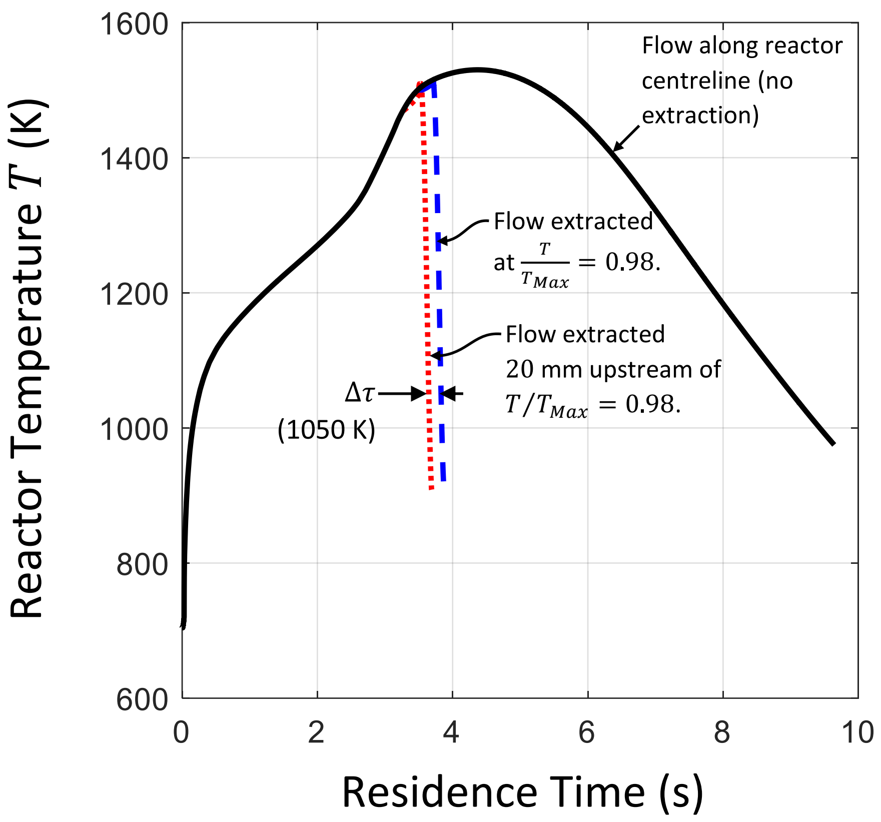

Figure S3: Predicted temperature profile and residence time of flow within the reactor along the centreline absent sampling tube, and for when sampling tube is performed within the zone of rising temperature.

To clarify the location of the reactor from where the sampling takes place, plots of the streamlines of flow that enters the sampling tube are given in Figure S4. It is evident that all sampled flow is drawn from upstream of the sampling tube inlet, and does not recirculate within the reactor before it is sampled. From simulation, the drop in the pressure of flow upon entry to the sampling tube is approximately 1.46 Pa.

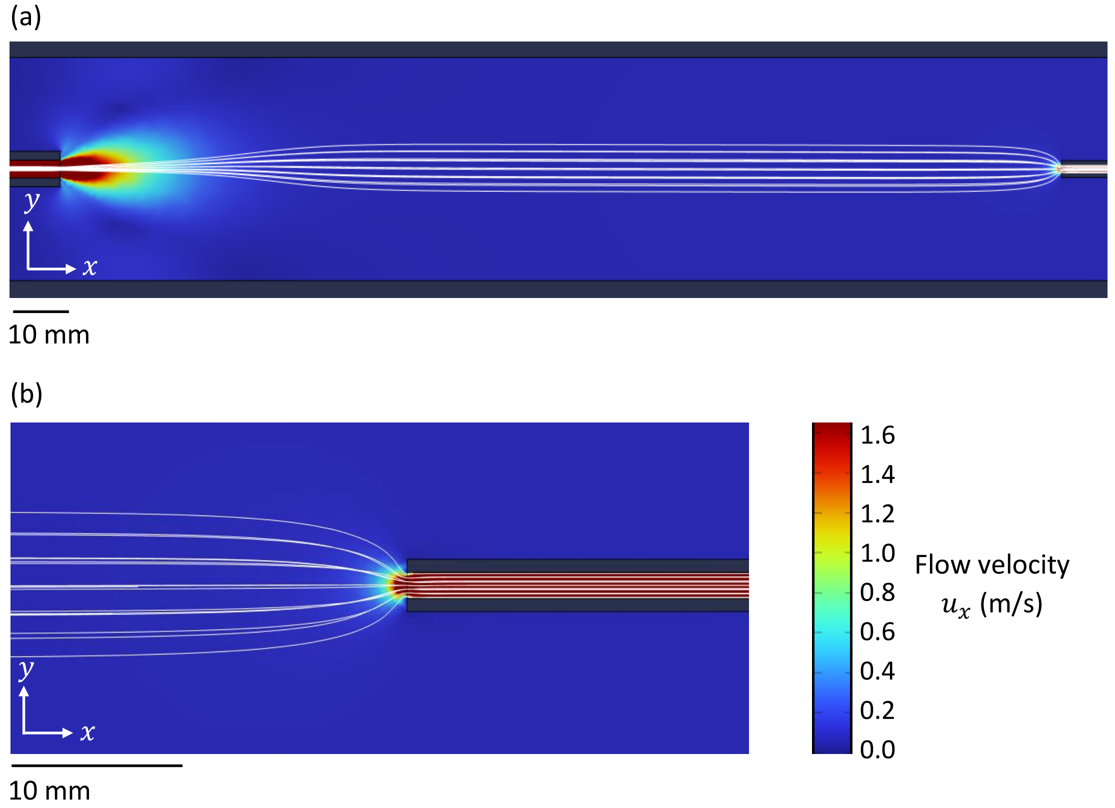

Figure S4: Simulation of flow (a) within a reactor before entry to the sampling tube, and (b) streamlines at the point of entry and along the sampling tube.

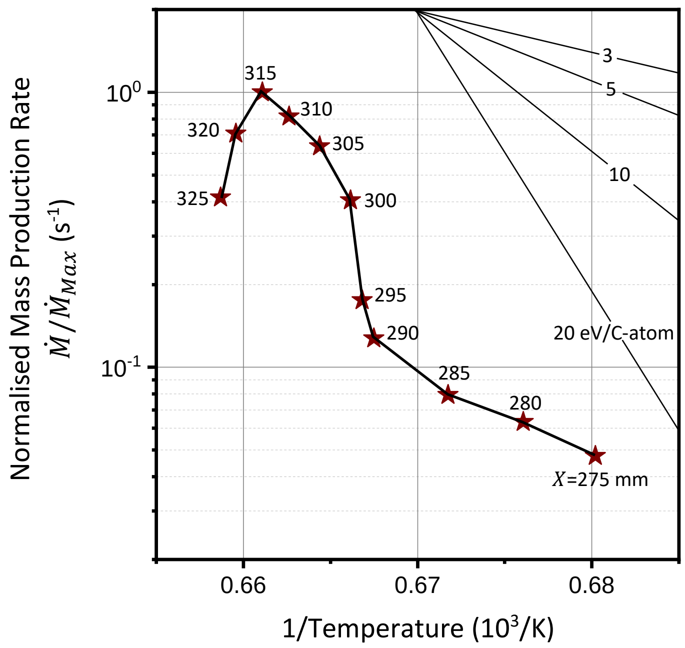

Figure S5: Arrhenius plot charting the rate of mass production in the zone of rising temperature within the range of position 275 mm $\leq X \leq$ 325 mm, plotted against the variation in reactor temperature.
